# CCL5 promotes LFA-1 expression in Th17 cells and induces LCK and ZAP70 activation in a mouse model of Parkinson’s disease

**DOI:** 10.3389/fnagi.2023.1250685

**Published:** 2023-11-03

**Authors:** Jingwei Zhao, Ke An, Zhijuan Mao, Yi Qu, Danlei Wang, Jiangting Li, Zhe Min, Zheng Xue

**Affiliations:** Department of Neurology, Tongji Hospital, Tongji Medical College, Huazhong University of Science and Technology, Wuhan, China

**Keywords:** Parkinson’s disease, Th17, CCL5, LFA-1, plck, pZAP70

## Abstract

**Background:**

Parkinson’s disease (PD), which is associated to autoimmune disorders, is characterized by the pathological deposition of alpha-synuclein (α-Syn) and loss of dopaminergic (DA) neurons. Th17 cells are thought to be responsible for the direct loss of DA neurons. C-C chemokine ligand 5 (CCL5) specifically induces Th17 cell infiltration into the SN. However, the specific effect of CCL5 on Th17 cells in PD and the relationship between CCL5 and lymphocyte function-associated antigen-1 (LFA-1) expression in Th17 cells are unknown.

**Methods:**

We evaluated the effects of CCL5 on LFA-1 expression in Th17 cells in mice treated with 1-methyl-4-phenyl-1,2,3,6-tetrahydropyridine (MPTP) and examined Th17 cell differentiation upon CCL5 stimulation *in vitro*. Furthermore, we assessed the effects of CCL5 on tyrosine kinase zeta-chain-associated protein kinase 70 (ZAP70) and lymphocyte-specific protein tyrosine kinase (LCK) activity in CCL5-stimulated Th17 cells *in vivo* and *in vitro*.

**Results:**

CCL5 increased the proportion of peripheral Th17 cells in MPTP-treated mice, LFA-1 expression on Th17 cells, and Th17 cell levels in the SN of MPTP-treated mice. CCL5 promoted Th17 cell differentiation and LFA-1 expression in naive T cells *in vitro*. Moreover, CCL5 increased Th17 cell differentiation and LFA-1 expression by stimulating LCK and ZAP70 activation in naive CD4^+^ T cells. Inhibiting LCK and ZAP70 activation reduced the proportion of peripheral Th17 cells and LFA-1 surface expression in MPTP-treated mice, and Th17 cell levels in the SN also significantly decreased.

**Conclusion:**

CCL5, which increased Th17 cell differentiation and LFA-1 protein expression by activating LCK and ZAP70, could increase the Th17 cell number in the SN, induce DA neuron death and aggravate PD.

## 1. Introduction

The second most common neurodegenerative disorder is Parkinson’s disease (PD) (Feigin et al., 2019). The typical symptoms of PD are motor symptoms, such as bradykinesia and tremor, and nonmotor symptoms, including reduced cognitive ability (Armstrong and Okun, 2020). The mechanism underlying PD involves the pathological accumulation of alpha-synuclein (α-Syn) in the central nervous system (CNS) and the loss of dopaminergic (DA) neurons in the substantia nigra pars compacta (SNpc) (Reich and Savitt, 2019). However, numerous studies have shown that Parkinson’s disease may be associated with autoimmune disorders, and that neuroinflammation is also closely related to the pathogenesis of PD (Tan et al., 2020; Grotemeyer et al., 2022). The number of CD4^+^ T cells in the SNpc is increased in individuals with PD, and when CD4^+^ cell infiltration is reduced, the number of DA neurons in the SNpc is maintained (Brochard et al., 2009). The infiltration of the SN by Th17 cells, which are a subgroup of CD4^+^ cells, is also increased in PD mice, and these cells are closely associated with the loss of DA neurons and the progression of PD (Siffrin et al., 2010; Liu et al., 2017). Therefore, Th17 cells may play a vital role in the pathological progression of PD.

As proinflammatory immune cells, Th17 cells play a role in neuroinflammation that cannot be ignored (Pikor et al., 2015). Studies have indicated that the proportion of Th17 cells in the peripheral blood of PD patients and PD mice is increased (Chen et al., 2015, 2017; Yang et al., 2017; Sommer et al., 2018), and the number of Th17 cells in the SN is increased in PD (Prots and Winner, 2019). Moreover, one study showed that Th17 cells in the SN use lymphocyte function-associated antigen-1 (LFA-1) to promote DA neuron death (Liu et al., 2017). Since the loss of DA neurons caused by Th17 cell infiltration of the SN is a nonnegligible component of the pathological mechanism underlying PD, preventing the infiltration of the SN by Th17 cells is critical for protecting against the loss of DA neurons.

Since the inhibition of Th17 infiltration of the SNpc is important for the protection of DA neurons, the mechanism underlying this process needs to be investigated. One study showed that T-cell entry into the CNS depend on surface expression of the LFA-1 protein (Glatigny et al., 2011; Rothhammer et al., 2011). Moreover, LFA-1 is the key molecule involved in the Th17 cell-mediated loss of DA neurons. LFA-1 can promote the death of DA neurons by binding surface ICAM-1 (Liu et al., 2017). Therefore, LFA-1 should not be ignored when investigating the mechanism underlying the Th17 cell-mediated neuroimmune inflammatory response.

C-C chemokine ligand 5 (CCL5) is an inflammatory chemokine that participates in inflammatory responses and chemokine signaling in inflammatory cells (Appay and Rowland-Jones, 2001). It effectively promotes the chemotaxis of Th17 cells toward sites of inflammation (Huffman et al., 2020). The roles of CCL5 in cancer and acquired immune deficiency syndrome (AIDS) have been widely studied. CCL5 promotes human immunodeficiency virus (HIV) replication through its natural receptor, CCR5, which is also used by HIV to enter T cells (Appay and Rowland-Jones, 2001). In addition, CCL5 can promote the formation of blood vessels in tumors, the migration of T cells to tumors and T-cell invasion of tumors through CCR5 receptors on the surface of T cells (Korbecki et al., 2020). In patients with PD, the serum concentration of CCL5 has been shown to be increased and positively correlated with PD severity (Tang et al., 2014). Moreover, it has been reported that CCL5 can specifically induce Th17 cell migration into the SN, resulting in the loss of DA neurons by constructing mouse models of PD (Dutta et al., 2019). However, when CCL5 activity was inhibited in the PD model mice, the number of Th17 cells in the SN was significantly decreased, and pathological damage due to PD was substantially improved (Chandra et al., 2016). Therefore, the effects of CCL5 on Th17 cells cannot be ignored in investigations of the pathogenesis and progression of PD. However, the specific effects of CCL5 on Th17 cells and the underlying mechanism involved in PD have not been clarified.

Thus, CCL5 strongly induces the infiltration of the SNpc by Th17 cells; the main mechanism through which Th17 cells migrate to and infiltrate the SNpc involves the LFA-1 protein. Studies have shown that CCL5 can stimulate the expression of LFA-1 on T cells (Szabo et al., 1997). Additionally, the activation of CCR5, the natural receptor of CCL5, can promote the expression of LFA-1 on T cells (Mukai et al., 2001). However, the relationship between CCL5 and LFA-1 expression on the Th17 cell surface in the context of PD has not been clarified.

In this study, we examined changes in the proportion of spleen Th17 cells and surface LFA-1 levels on Th17 cells in 1-methyl-4-phenyl-1,2,3,6-tetrahydropyridine (MPTP)-treated mice and MPTP-and CCL5-treated mice, as well as the loss of DA neurons caused by Th17 cell infiltration of the SN. We also examined the effect of CCL5 on Th17 cells *in vitro*. In addition, we investigated the mechanism by which CCL5 induces LFA-1 expression *in vivo* and *in vitro*. We demonstrated that CCL5 promoted not only an increase in the proportion of splenic Th17 cells but also LFA-1 expression on Th17 cells, which was conducive to their infiltration of the SN, resulting in the loss of DA neurons. The effect of CCL5 on Th17 migration was found to be mediated by the phosphorylation of lymphocyte-specific protein tyrosine kinase (LCK) and zeta-chain associated protein kinase 70 (ZAP70). Our findings provide new evidence that helps to clarify the pathogenesis of PD and may aid in the development of a clinical treatment.

## 2. Materials and methods

### 2.1. Animal model

The male C57BL/6J mice (Beijing HFK Bioscience, Beijing, China) used in this study were 7–8 weeks old, and the body weight of each mouse was ≤20 g. All mice were housed in a specific pathogen-free (SPF) animal room at Tongji Medical College on a 12/12 h light/dark cycle. All animal procedures conformed to the Animal Research: Reporting of *In Vivo* Experiments (ARRIVE) guidelines and were carried out in accordance with the U.K. Animals (Scientific Procedures) Act, 1986 and associated guidelines, EU Directive 2010/63/EU for animal experiments, and the National Research Council’s Guide for the Care and Use of Laboratory Animals.

### 2.2. MPTP treatment

Mice were administered four intraperitoneal injections of phosphate-buffered saline (PBS; 10 mL/kg body weight) or MPTP (20 mg/kg body weight, Sigma, United States, 23007-85-4) every two hours. After the last injection of phosphate-buffered saline (PBS) or MPTP, the mice in the PBS/MPTP group were randomly divided into two groups: one group was intraperitoneally injected with PBS, and the other group received an intraperitoneal injection of mouse recombinant cytokine CCL5 (PeproTech, 250-07, 5 μg/kg). The treatments were administered on day 3 and day 6. Each mouse received an intraperitoneal injection of 100 μL of PBS or 100 μL of a CCL5 solution (100 ng of CCL5 dissolved in 100 μL of PBS). On the day after the last injection of CCL5, the mice were sacrificed, and the brains and spleens were processed for follow-up experiments and analyses.

### 2.3. Cell purification and culture

Naive CD4^+^ T cells were directly extracted from the spleens of mice using a naive CD4^+^ T-cell isolation kit for mice (Miltenyi Biotec, 130-104-453) for *in vitro* culture and to induce differentiation. Roswell Park Memorial Institute-1640 (RPMI-1640) medium that contained 10% T-cell-specific fetal bovine serum (FBS), 1% GlutaMax, 1% HEPES and 1% penicillin–streptomycin (all from Gibco, Shanghai, China) was used to culture the purified T cells. The culture plates were precoated with anti-CD3 (10 μg/mL, BD Biosciences, 553057) and anti-CD28 (20 μg/mL, BD Biosciences, 553294) antibodies for 12–16 h. In general, we used the cytokines TGF-β (10 ng/mL, MCE, HY-P7117), IL-23 (10 ng/mL, R&D Systems, United States, 1887-ML) and IL-6 (10 ng/mL, MCE, HY-P7063) to induce naive CD4^+^ T cells to differentiate into Th17 cells. TGF-β and IL-2 (10 ng/mL, MCE, HY-P7077) were used to induce naive CD4^+^ T cells to differentiate into Treg cells; however, IL-2 and IL-12 (10 ng/mL, MCE, HY-P7315) were used to induce naive CD4^+^ T cells to differentiate into Th1 cells. Purified naive CD4^+^ T cells were cultured in an incubator containing 5% CO_2_ for further study.

### 2.4. Behavioral testing

The ability of the mice to maintain balance and coordination in a rotating rod apparatus (IITC Life Science) was evaluated. The rotating rod device featured a long rod that could be turned by an electric motor, and the apparatus contained separate compartments for each mouse. The mice were placed perpendicular to a long rod on a rotating shaft, and their heads were turned in the direction of rotation. For this test, the mice underwent a three-day training and adaptation period during which the rod rotation device was set at 20 rpm for a duration of 10 min. At the end of the training period, the ability of the mice to balance and coordinate on the rotating rod was tested. The equipment parameters used during testing were the same as those used during training. Each mouse was tested three times, and the time spent on the rotary rod was recorded. To mitigate the effects of stress and fatigue, tests performed using the same mice were administered at least 5 min apart.

In addition, the ability of the mice to maintain balance and coordination on a pole (50 cm in height and 1 cm in diameter) was tested. The mice were placed upside down at the top of the pole, and the time that it took to descend the pole was recorded. Each mouse was evaluated three times and trained to adapt to the device 3 days in advance.

### 2.5. Flow cytometry

Flow cytometry was performed to characterize the proportions of CD4^+^ T cells and Th17 cells. The levels of CCR5, LFA-1, pZAP70 and pLCK were measured. To block the secretion of extracellular proteins, T cells were stimulated with 20 ng/mL leukocyte activation cocktail (BD Biosciences, 550583) for 4–6 h. Then, the cells were collected and washed with PBS. The cells were maintained in flow staining buffer containing a diluted FITC-labeled anti-CD4 antibody (5 μg/mL, BD Biosciences, 553729), BV421-labeled anti-CCR5 antibody (5 μg/mL, BD Biosciences, 743695), PE-labeled anti-CCR5 antibody (2 μg/mL, BioLegend, 107005), APC-labeled anti-LFA-1 antibody (2 μg/mL, BioLegend, 141009), FITC-labeled anti-TCR antibody (5 μg/mL, BD Biosciences, 553170), and APC-labeled anti-CD4 antibody (2 μg/mL, BioLegend, 100516) for 30 min at 4°C. The cells were then resuspended in fixation and permeabilization buffer for 45 min. After 45 min of incubation in the dark, the cells were incubated with a diluted PE-labeled antibody targeting the Th17-specific marker IL-17A (5 μg/mL, BD Biosciences, 559502), PE-labeled antibody targeting pLCK (1:50, Cell Signaling Technology, 14791S), PE-labeled antibody targeting pZAP70 (1:50, Cell Signaling Technology, 94361S), PEcy7-labeled antibody targeting IFN-gamma (5 μg/mL, Invitrogen, 2153357) or PEcy7-labeled antibody targeting Foxp3 (5 μg/mL, Invitrogen, 2378858) at 4°C in the dark for another 45 min. A BV421-labeled antibody targeting the Th17-specific marker IL-17A (5 μg/mL, BioLegend, 506925) was used to perform pZAP70 or pLCK staining. After incubation, the cell suspension was centrifuged, washed once and then suspended in 1% paraformaldehyde. The cells were then analyzed using Cytoexpert (BD Biosciences). Cells were gated based on morphological characteristics. All cell analyses were performed using CD4^+^ lymphocyte populations.

### 2.6. Western blotting

Western blotting was performed according to standard procedures. Briefly, the mice were sacrificed, and the SN was collected. Tissues were immersed in radioimmunoprecipitation assay (RIPA) buffer (Servicebio, Wuhan, China) containing a protease inhibitor cocktail (Servicebio, Wuhan, China). Then, the buffer containing tissue fragments was centrifuged at 12,000 × g for 10 min at 4°C. After centrifugation, the supernatant containing the dissolved proteins was collected. Equal amounts of proteins were transferred onto PVDF membranes after electrophoresis on 10% SDS–PAGE gels. The blots were incubated with anti-ICAM-1 (1:1000, Abcam, ab222736), anti-TH (1:1000, Abcam, ab137869), anti-Ror gamma(t) (1:1000, Thermo Fisher, 14-6988-80), anti-α-Syn (1:1000, Cell Signaling Technology, 2644S), anti-H3 (1:1000, ABclonal, A2348), anti-GAPDH (1:50000, Proteintech, 60004-1-lg), anti-BAX (1:1000, ABclonal, A20227), anti-Bcl-2 (1:1000, ABclonal, A21592), anti-ZAP70 (1:1000, ABclonal, A9536), anti-LCK (1:1000, ABclonal, A2177), anti-CD11a (1:1000, Abcam, ab228964), anti-pZAP70 (1:1000, Bioworld, BS4884) and anti-pLCK (1:1000, Affinity, AF3101) antibodies for 12–16 h at 4°C. Then, the blots were incubated at room temperature (RT) with secondary antibodies (1:5000, Servicebio) chosen based on the species from which the primary antibodies were derived. Finally, the blots were scanned with an exposure apparatus. Band intensities were quantified using ImageJ software (NIH, United States).

### 2.7. Immunofluorescence and counting of DA neurons in the SN

The mouse brains were fixed with 4% paraformaldehyde after sacrifice. SNpc tissue was embedded in paraffin and sectioned. Coronal sections (30 μm) were cut from the midbrain region and processed for immunostaining. The SNpc tissue sections were blocked for 1 h with 10% bovine serum albumin (BSA) in PBS. Then, the samples were kept in a mixture of several primary antibodies [antibodies specific for TH (1:200, mouse origin, Abcam, ab137869) and BAX (1:50, ABclonal, A20227) or CCR5 (1:50, ABclonal, A20261), CD4 (1:50, rat origin, BioLegend, 100505) or IL-17A (1:40, rabbit origin, Affinity, DF6127)] diluted in blocking solution. Then, the samples were incubated at 4°C for 12–16 h. The next day, PBS was used to wash the samples for 15 min, and the samples were then incubated with secondary antibodies (all 1:200; Yeasen) for 1 h in the dark. Following 15 min washes with PBS, the samples were treated with an anti-tissue fluorescence-quenching agent containing DAPI and covered with a cover glass. Then, the samples were stored in the dark at 4°C.

### 2.8. Statistical analysis

GraphPad Prism v7.0 was used for statistical analysis of the data. Values are expressed as the mean ± standard error of the mean (SEM). student’s *t*-test was used to assess the statistical significance of differences between two different samples. Multiple samples were compared using analysis of variance (ANOVA), followed by Tukey’s multiple comparison test. A *p*-value of <0.05 was used as the criterion for statistical significance.

## 3. Results

### 3.1. CCL5 aggravated MPTP-induced PD-like manifestations and the loss of DA neurons in the SNpc in mice

**Figure 1:**
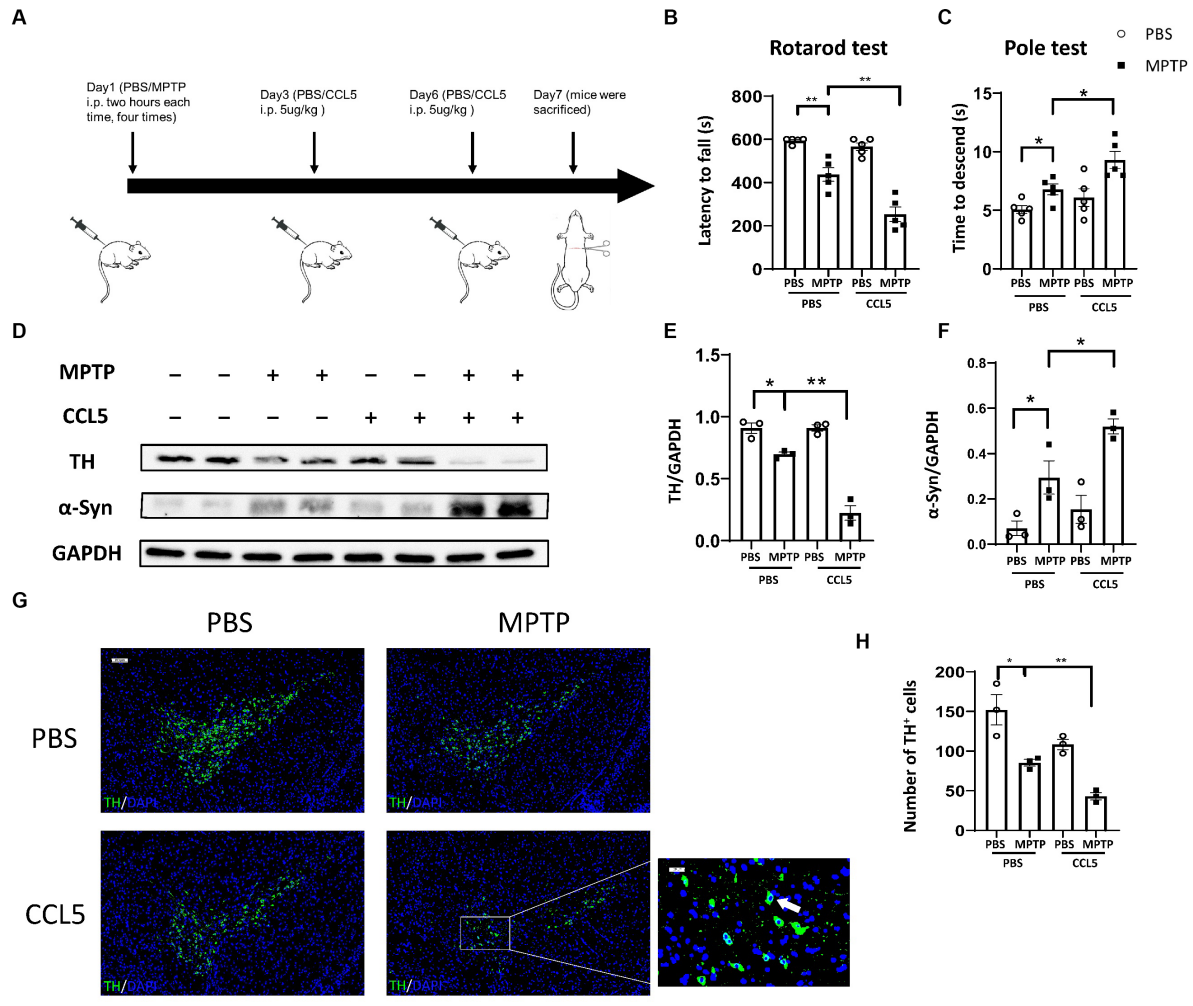
Behavioral changes and pathological changes in MPTP-injected mice were more severe after CCL5 treatment. **(A)** The timeframe of the mouse treatments. **(B)** The period over which the mouse maintained its position on the rotating rod was defined as the latency to fall in the rotarod test *n* = 5 per group. **(C)** The time needed for mice to descend from the top to the bottom of a pole was defined as the time to descend in the pole test *n* = 5 per group. **(D–F)** Western blot analysis was used to measure TH and α-Syn levels in the brain SNpc of the mice, and the data were quantified using ImageJ *n* = 3 per group. **(G,H)** Immunofluorescence was used to count DA neurons, which were stained for TH. The arrows within the enlarged area of interest indicated DA neurons in the SNpc, as shown by immunofluorescence staining. The DA neurons in the SNpc were counted using ImageJ and GraphPad software *n* = 3 per group. Unenlarged images scale bars = 200 μm. Image scale bar of the enlarged area of interest = 50 μm. Data are presented as the means ± SEMs. ^*^p < 0.05 and ^**^p < 0.01.

To establish an animal model, we referred to previous work (Dutta et al., 2019) and treated mice with MPTP and CCL5 (Figure 1A). To investigate the effect of CCL5 on MPTP-treated mice, we first assessed behavioral changes in the mice. The MPTP-injected mice exhibited a shorter duration on the rotating rod than the control mice (Figure 1B). Additionally, the MPTP-injected mice treated with CCL5 spent less time on the rotating rod than the MPTP-injected mice that were not treated with CCL5 (Figure 1B). Moreover, the results of the pole test suggested that the time for the mice to descend the pole was longer in the MPTP group than in the PBS group, while the time for the mice to descend the pole in the CCL5 and MPTP groups were longer than that of mice in the MPTP group (Figure 1C). In summary, after MPTP injection for PD modeling, the mice showed behavioral deficits that were exacerbated by CCL5, as indicated by the longer time to descend the pole.

To determine the effect of CCL5 on neurodegeneration in MPTP-treated mice, we measured the expression levels of TH and pathological α-Syn deposits in the SN. TH expression in the SN of the MPTP-treated mice significantly decreased, while that in the CCL5-treated MPTP-injected mice decreased even more significantly (Figures 1D,E). In addition, the BAX/Bcl2 ratio, which can reflect apoptosis, in the SNpc was significantly higher in the MPTP group than in the control group, and the ratio in the SNpc was significantly higher in the CCL5-treated MPTP group than in the MPTP-treated mice that were not treated with CCL5 (Supplementary Figure S1A). Furthermore, α-Syn deposition in the SN of the CCL5-treated MPTP-injected mice was greater than that in the MPTP-injected mice that did not receive CCL5 treatment; additionally, α-Syn deposition in the MPTP-treated mice was greater than that in the PBS-treated mice (Figures 1D,F). More importantly, the number of DA neurons in the SNpc was significantly decreased in the MPTP-treated mice and further decreased in the CCL5-treated MPTP-injected mice (Figures 1G,H). The immunofluorescence staining results also confirmed that the proportion of apoptotic cells increased. The percentage of TH^+^BAX^+^ cells in the SNpc was significantly higher in the MPTP-treated mice than in the control mice and even higher in the CCL5-treated mice than in the MPTP-treated mice (Supplementary Figures S1C,D). These results suggest that CCL5 could aggravate the MPTP-induced loss of DA neurons.

### 3.2. CCL5 induced an increase in not only the ratio of Th17 cells and surface LFA-1 protein expression but also the number of Th17 cells in the mouse SNpc

**Figure 2:**
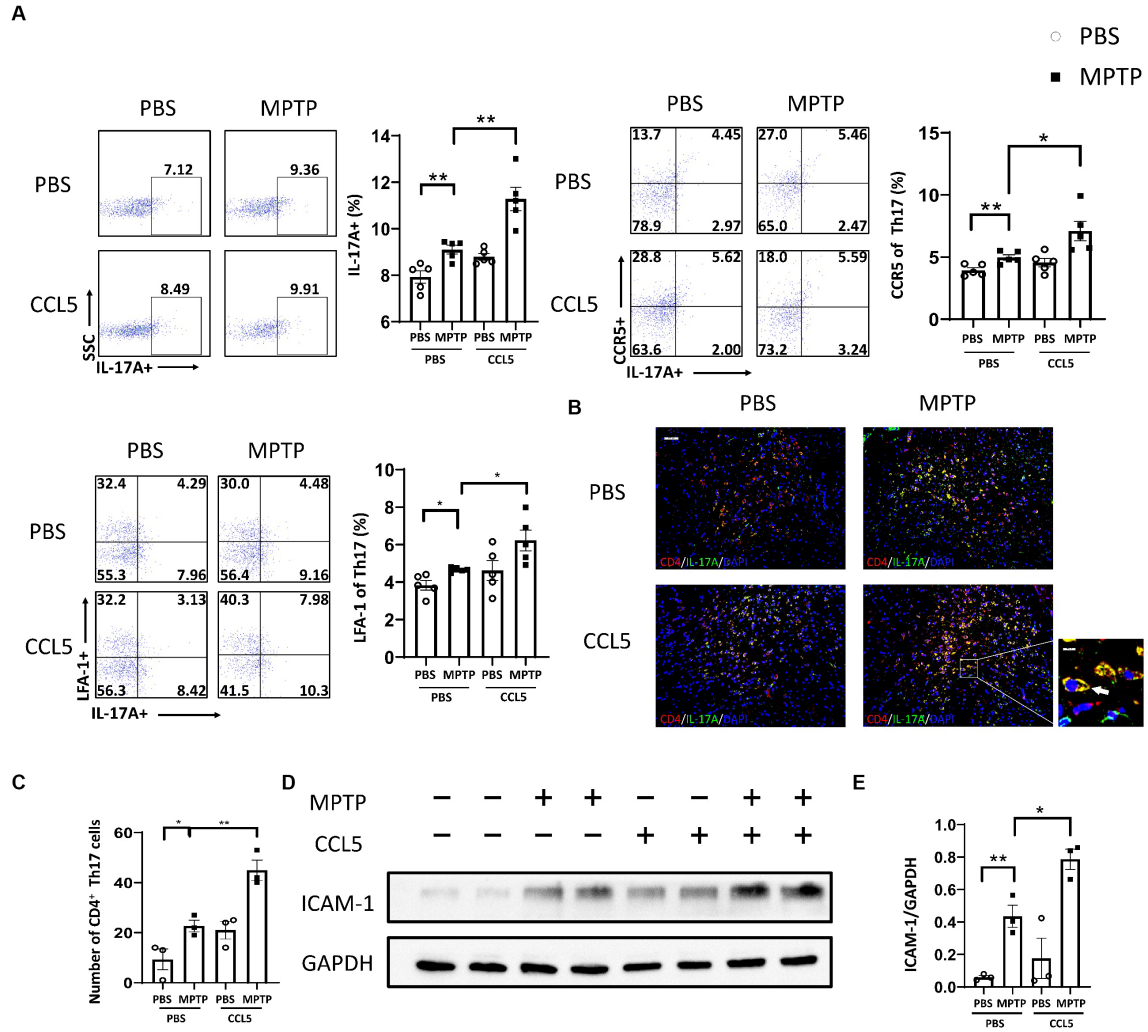
CCL5 promoted not only an increase in the peripheral Th17 ratio and LFA-1 expression but also the loss of DA neurons in the SNpc and the binding of DA neurons by Th17 cells. **(A)** Flow cytometry and FlowJo software were used to analyze peripheral PBMCs from mice. The proportions of IL-17A^+^, IL-17A^+^ and CCR5^+^, IL-17A^+^ and LFA-1^+^ cells among CD4^+^ cell populations were calculated *n* = 5 per group. **(B,C)** Immunofluorescence was used to count CD4^+^ Th17 cells in the SNpc, which were stained for CD4 and IL-17A. A cell was identified as a CD4^+^ Th17 cell when both stained CD4 and IL-17A were present around the nucleus stained by DAPI and took on a cytoplasmic form that completely or partially encircled the nucleus. The arrows within the enlarged area of interest indicated CD4^+^ Th17 cells in the SNpc, as shown by immunofluorescence staining. The CD4^+^ Th17 cells in the SNpc were counted using ImageJ and GraphPad software *n* = 3 per group. Unenlarged images scale bars = 100 μm. Image scale bar of the enlarged area of interest = 20 μm. **(D,E)** Western blot analysis was used to detect ICAM-1 in the brain SNpc of mice, and the data were quantified using ImageJ *n* = 3 per group. Data are presented as the means ± SEMs. ^*^p < 0.05 and ^**^p < 0.01.

Since CCL5, an inflammatory factor in peripheral blood, can affect peripheral T cells, we examined changes in Th17, Th1, and Treg cells after the stimulation of MPTP-treated mice with CCL5. The proportion of Th17 cells in the spleen of the MPTP-treated mice was significantly higher than that in the control mice, and the number of Th17 cells in the spleen was higher in the mice treated with MPTP and CCL5 than in the MPTP-treated mice (Figure 2A). However, although the proportion of Th1 cells in the spleen was higher in the MPTP-treated mice than in the control group, CCL5 did not promote a further increase in the proportion of Th1 cells in the spleen of the MPTP-treated mice (Supplementary Figure S2A). In addition, although CCL5 treatment increased the proportion of splenic Treg cells, which was decreased in MPTP-treated mice, it did not promote the expression of LFA-1 on Treg cells (Supplementary Figures S2B,C). In contrast, the expression levels of CCR5, the receptor of CCL5, and LFA-1, which helps Th17 cells migrate to the SN, were increased on the surface of Th17 cells in the spleens of mice after CCL5 treatment (Figure 2A). In summary, CCL5 did not affect the proportion of peripheral Th1 cells or the expression of LFA-1 on the Treg cell surface; however, it increased not only the Th17 cell differentiation ratio in the spleen of the mice but also the expression of the CCR5 receptor and LFA-1 on the surface of Th17 cells.

Combined with the previous results, these findings indicate that CCL5 aggravated the loss of DA neurons in the SNpc and increased the proportion of peripheral Th17 cells in MPTP-treated mice. We used immunofluorescence staining to detect the number of Th17 cells in the SNpc to determine whether CCL5 exacerbated the loss of DA neurons by affecting the number or function of Th17 cells. The number of Th17 cells in the SN was increased in the MPTP-treated mice and further increased in the CCL5-treated MPTP-injected mice (Figures 2B,C). More importantly, the number of CCR5-expressing Th17 cells in the SNpc was higher in the MPTP-treated mice that were treated with CCL5 than in the MPTP-treated mice that were not treated with CCL5 (Supplementary Figures S2D–F). This suggests that CCL5 promoted the expression of CCR5 on Th17 cells in the SNpc. As previously proposed, Th17 cells may contact DA neurons through the LFA-1/ICAM-1 pathway to induce cell death (Liu et al., 2017). We measured ICAM-1 levels in the SNpc of mice to determine the amount of DA neuron loss caused by Th17 cells. ICAM-1 expression was found to be increased in the mice in the MPTP group, while the expression level of ICAM-1 in the CCL5-treated MPTP-injected mice was higher than that in the MPTP group (Figures 2D,E). This finding suggests that CCL5 could promote an increase in the number of Th17 cells in the SN of PD mice and promote DA neuron death by activating ICAM-1 on the surface of DA neurons.

**Figure 3:**
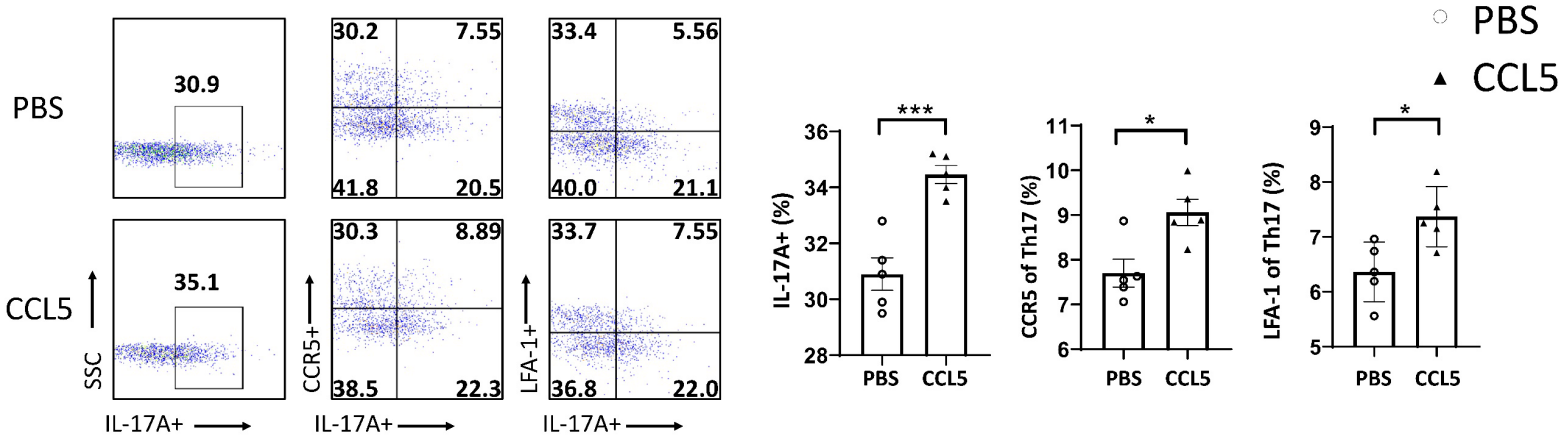
CCL5 promoted naive CD4^+^ T-cell differentiation into Th17 cells and the expression of CCR5 and LFA-1. Naive CD4^+^ T cells from the spleen of C57BL/6J mice were purified by magnetic beads *in vitro* and induced to differentiate into Th17 cells by PBS/CCL5 (1 μg/mL) treatment. After 3 days, the cells were collected. Flow cytometry and FlowJo software were used to analyze the cells. The proportions of IL-17A^+^, IL-17A^+^ and CCR5^+^, IL-17A^+^ and LFA-1^+^ cells among CD4^+^ cell populations were calculated *n* = 5 per group. Data are presented as the means ± SEMs. ^*^p < 0.05 and ^***^p < 0.001.

To further clarify the effect of CCL5 on Th17, Treg and Th1 cells, we purified naive CD4^+^ T cells and induced their differentiation into Th17, Th1, and Treg cells using different cytokines. All cells were cultured on cell culture plates precoated with anti-CD3 and anti-CD28 antibodies. Flow cytometry showed that the differentiation of Th17 cells was significantly increased (Figure 3). The levels of CCR5 and LFA-1 were also significantly increased (Figure 3). These results suggest that CCL5 could directly induce Th17-polarized differentiation and promote the expression of LFA-1. However, the proportion of Th1 cells upon stimulation with CCL5 did not significantly differ from that in the control group (Supplementary Figure S3A). Although the differentiation ratio of CCL5-stimulated Treg cells was lower than that in the control group, there was no significant difference in the expression of LFA-1 on the surface (Supplementary Figure S3A). The results of the *in vitro* experiments were consistent with those obtained in mice.

Based on the above data, CCL5 could increase the number of Th17 cells in the spleen and in the SNpc of PD mice and promote the expression of LFA-1 on the surface of Th17 cells to facilitate the extravascular dissociation of Th17 cells. Therefore, CCL5 may induce Th17 cell migration to the SNpc by promoting the expression of LFA-1 on the surface of Th17 cells and increasing the number of Th17 cells in the SNpc. The increased infiltration of the SN by Th17 cells could lead to inflammation in DA neurons and the exacerbation of PD.

### 3.3. CCL5 increased the proportion of Th17 cells and LFA-1 expression by activating LCK and ZAP70

**Figure 4:**
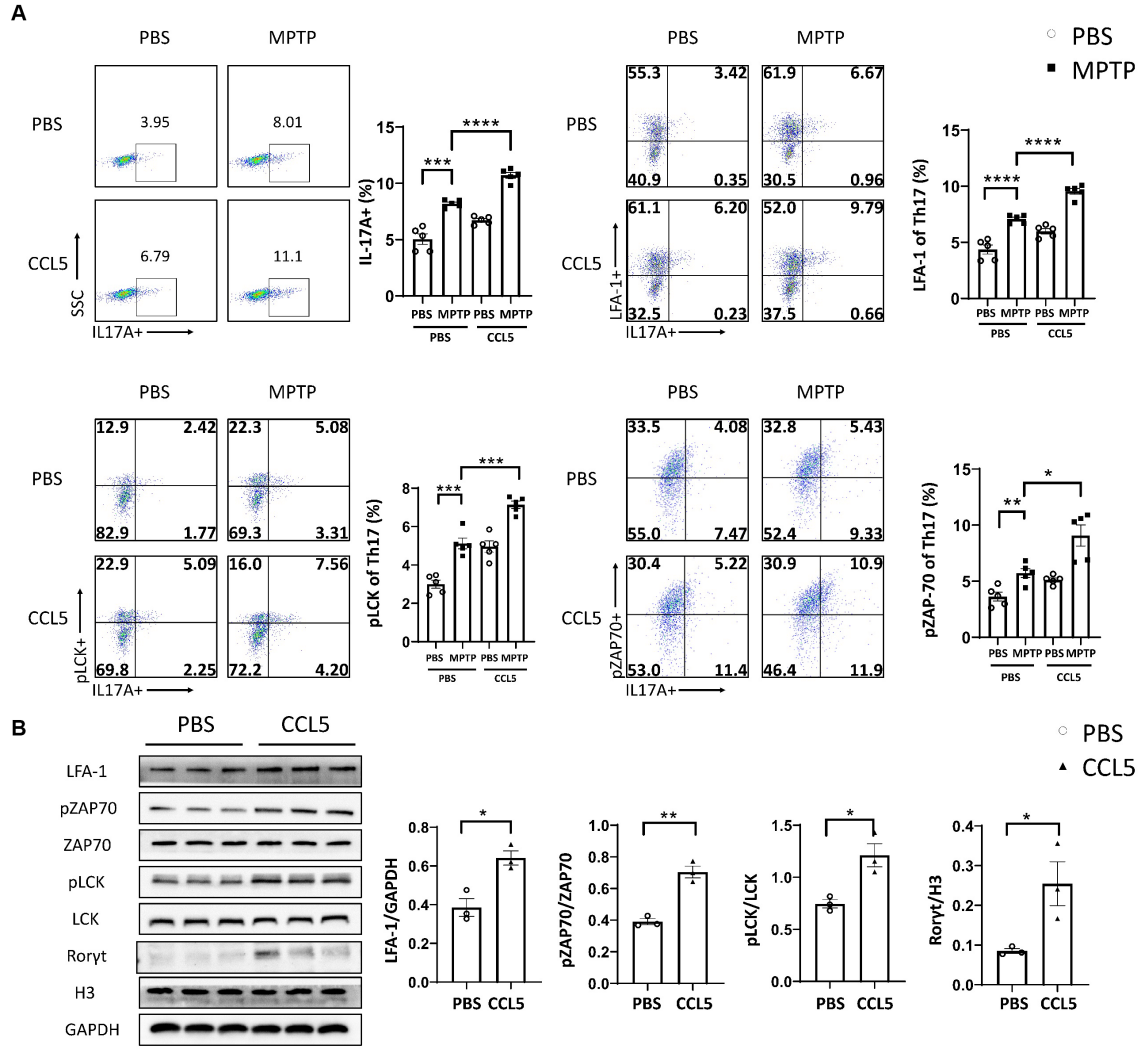
CCL5 promoted Th17 cell differentiation and LFA-1 expression by activating LCK and ZAP70. **(A)** Flow cytometry and FlowJo software were used to analyze peripheral PBMCs from mice. The proportions of IL-17A^+^, IL-17A^+^ and LFA-1^+^, IL-17A^+^ and pLCK^+^, IL-17A^+^ and pZAP70^+^ cells among CD4^+^ cell populations were calculated *n* = 5 per group. **(B)** Naive CD4^+^ T cells that were cultured *in vitro* were induced to differentiate into Th17 cells, which were treated with CCL5 or PBS. After 3 days, LFA-1, pLCK, pZAP70, total LCK, total ZAP70 and Rorγt levels were measured by Western blot analysis and quantified using ImageJ *n* = 3 per group. Data are presented as the means ± SEMs. ^*^p < 0.05, ^**^p < 0.01, ^***^p < 0.001, and ^****^p < 0.0001.

To investigate whether CCL5 induced changes in Th17 cell differentiation and function by activating ZAP70 and LCK, we measured the levels of pLCK and pZAP70. We found that the proportion of peripheral Th17 cells in the CCL5-treated MPTP-injected mice was higher than that in the MPTP-treated mice (Figure 4A), and the expression of LFA-1 on peripheral Th17 cells was also higher in the CCL5-treated MPTP-injected mice than in the MPTP-injected mice (Figure 4A). These results are consistent with the trend we describe in the Results section 3.2 of this paper. We also found that the levels of pLCK and pZAP70 were increased in Th17 cells isolated from MPTP-treated mice, while CCL5-treated mice showed even higher levels of these markers than MPTP-treated mice (Figure 4A). As proteins that promote Th17 cell differentiation and LFA-1 expression (Dutta et al., 2017), the increased proportions of pLCK and pZAP70 proteins in mouse spleen Th17 cells are interesting and worthy of further investigation. However, because the pLCK and pZAP70 proteins are also important in the TCR pathway (Dutta et al., 2017), to determine whether CCL5 activated the TCR pathway, we measured the amount of TCRs on the surface of Th17 cells. The number of TCRs on the Th17 cell surface in the MPTP-treated mice was significantly increased, while CCL5 did not promote an increase in the number of TCRs on the Th17 cell surface in the MPTP-treated mice (Supplementary Figure S4A).

To determine the effect of CCL5 on LCK and ZAP70 in naive CD4^+^ T cells differentiating into Th17 cells *in vitro*, magnetic bead sorting was used. We found that the proportion of phosphorylated LCK and ZAP70 among total LCK and ZAP70 in naive CD4^+^ T cells stimulated by CCL5 *in vitro* was higher than that in Th17 cells stimulated by PBS (Figure 4B). We also found that *in vitro*, CCL5-stimulated naive CD4^+^ T cells had higher levels of Th17 cell differentiation and LFA-1 expression than the control group (Figure 4B). These results indicated that CCL5 stimulation alone could significantly increase the differentiation ratio of Th17 cells and LFA-1 expression on the cell surface. Moreover, these results indicated that CCL5 may promote Th17 cell differentiation by increasing the activation of ZAP70 and LCK. To exclude the influence of the TCR pathway on pLCK and pZAP70, we measured the expression level of TCRs on the surface of Th17 cells cultured *in vitro* and found that there was no significant difference upon CCL5 stimulation (Supplementary Figure S4B).

### 3.4. Inhibition of pLCK and pZAP70 activation reduced the number of Th17 cells in the SN of PD mice

**Figure 5:**
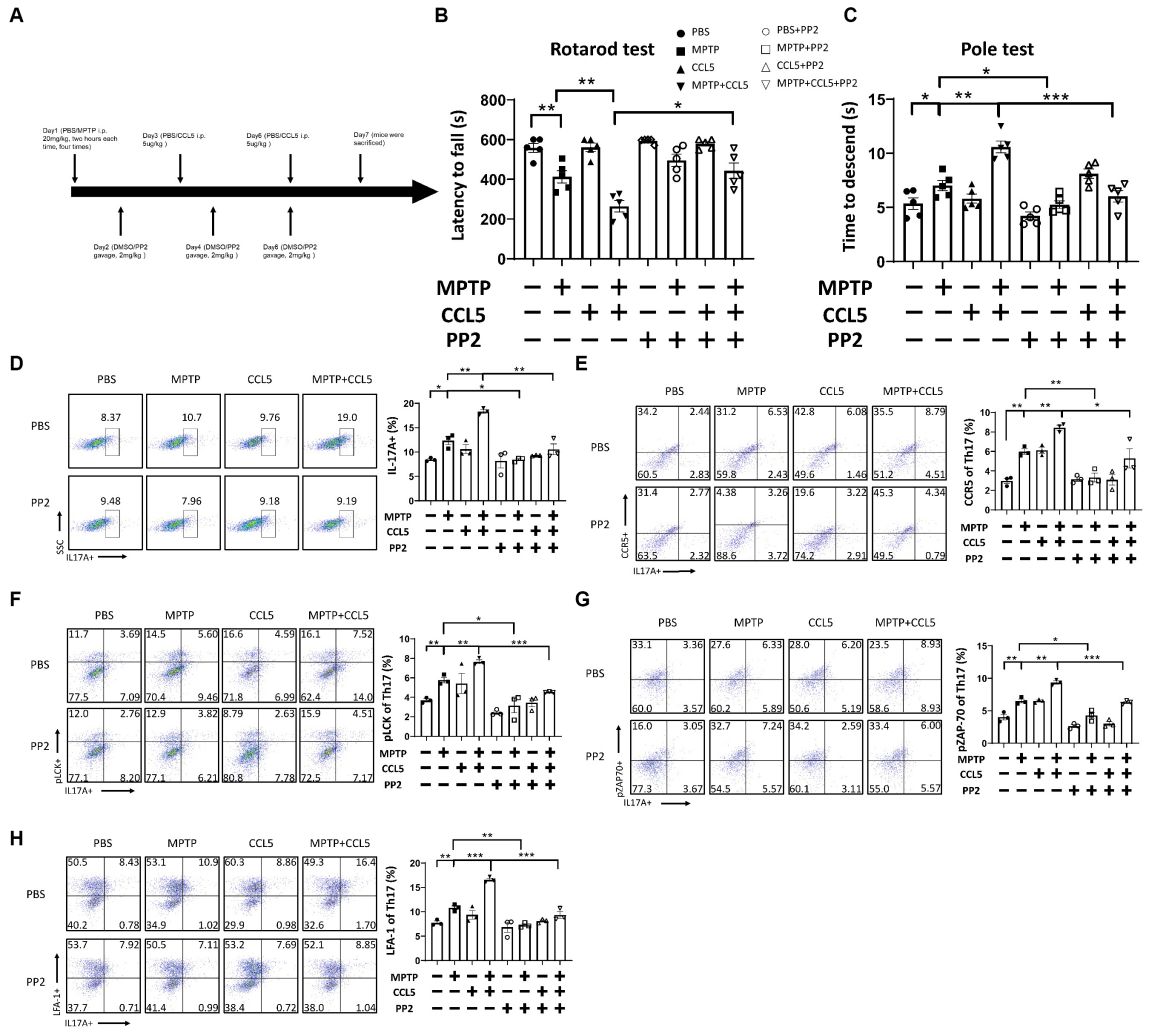
PP2 improved motor symptoms in mice, inhibited Th17 cell differentiation and reduced LFA-1 expression by inhibiting LCK and ZAP70 activation. **(A)** The timeline of the mouse treatment regimen. **(B)** Rotarod test. **(C)** Pole test. **(D–H)** After PP2 treatment, flow cytometry and FlowJo software were used to analyze peripheral PBMCs from the mice. The proportions of IL-17A^+^, IL-17A^+^ and CCR5^+^, IL-17A^+^ and LFA-1^+^, IL-17A^+^ and pLCK^+^, IL-17A^+^ and pZAP70^+^ cells among CD4^+^ cell populations were calculated *n* = 5 per group. Data are presented as the means ± SEMs. ^*^p < 0.05, ^**^p < 0.01, and ^***^p < 0.001.

The above results indicated a rapid increase in pLCK and pZAP70 levels in CCL5-treated Th17 cells. Therefore, to verify whether pLCK and pZAP70 are the key proteins through which CCL5 induces Th17 cell differentiation and increases LFA-1 expression on the surface of Th17 cells, we treated mice with PP2 (Figure 5A), which is an inhibitor of LCK activation, and measured the proportion of peripheral Th17 cells and LFA-1 expression on Th17 cells in the mice. When the inhibitor was administered, the behavioral deficits observed in the MPTP-injected and CCL5-treated mice were ameliorated. The mice treated with the inhibitor and both CCL5 and MPTP stayed on the rotating rod longer than the mice that were treated with both CCL5 and MPTP and did not receive the inhibitor (Figure 5B). Moreover, the MPTP-injected mice that received the inhibitor took less time to descend the pole to the ground than those that did not receive the inhibitor, and the mice that received the inhibitor and both CCL5 and MPTP showed the same reduction in time to descend the pole as those that did not receive the inhibitor but received MPTP and CCL5 (Figure 5C). After PP2 application, the proportions of Th17 cells and CCR5 receptors on Th17 cells in the spleen were significantly lower in the inhibitor-treated MPTP group and CCL5-treated MPTP group than in the same groups that did not receive inhibitor treatment (Figures 5D,E). Additionally, the pLCK and pZAP70 levels in Th17 cells were lower in the MPTP-treated mice and CCL5-treated, MPTP-injected mice than in the mice that received the same treatments that did not receive PP2 (Figures 5F,G). Most importantly, CCL5 failed to promote the expression of the LFA-1 protein on the Th17 cell surface in the CCL5-treated, MPTP-injected mice after the addition of PP2, and the level of LFA-1 on the Th17 cell surface was lower in the MPTP-injected mice treated with PP2 than in the MPTP-injected mice that did not receive inhibitor treatment (Figure 5H).

**Figure 6:**
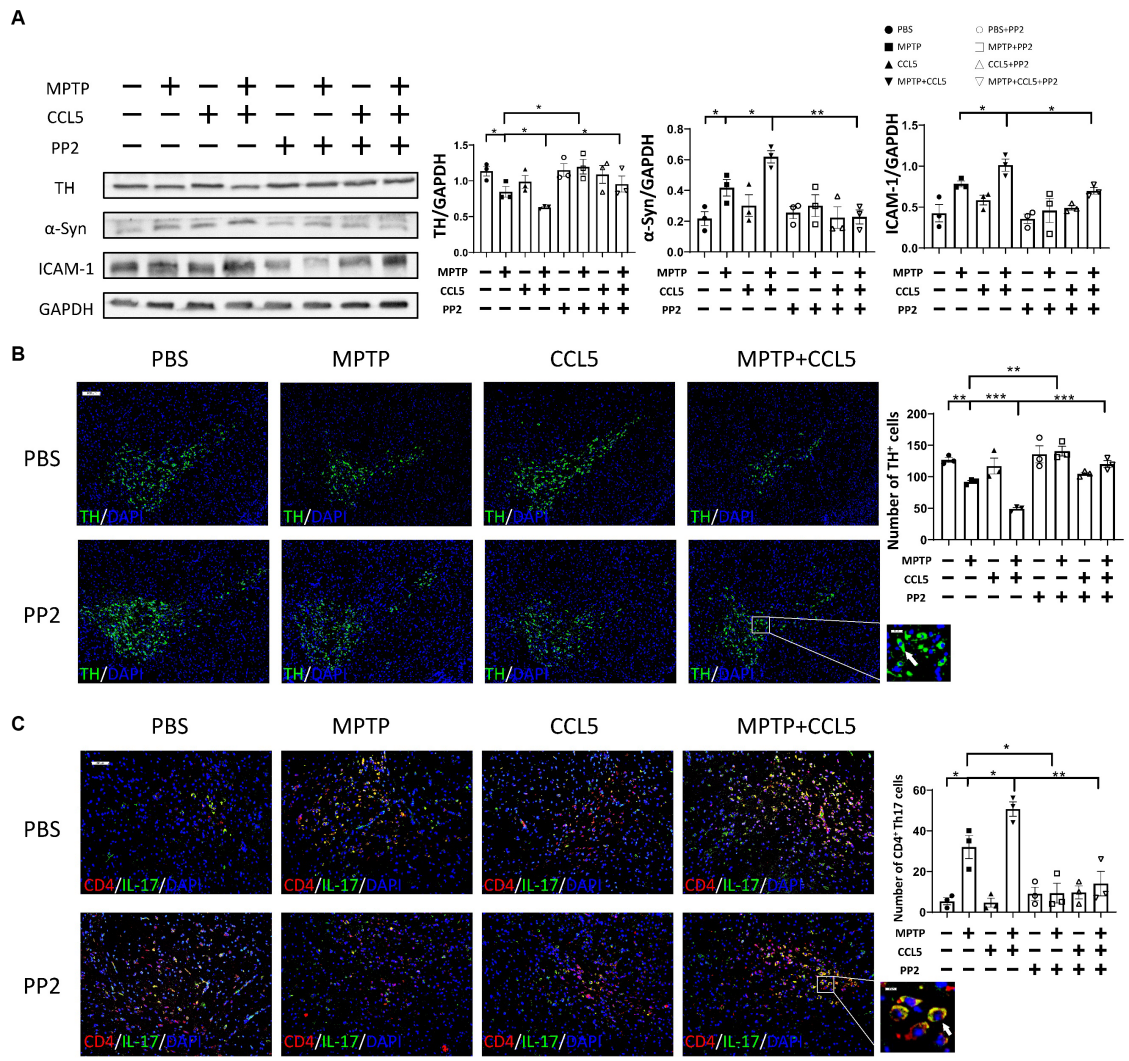
PP2 improved CCL5-induced pathological changes in the SN in MPTP-treated mice. **(A)** Western blot analysis was used to measure TH, ICAM-1 and α-Syn levels in the brain SNpc of mice, and the data were quantified using ImageJ *n* = 3 per group. **(B)** Immunofluorescence was used to count DA neurons, which were stained for TH. The arrows within the enlarged area of interest indicated DA neurons in the SNpc, as shown by immunofluorescence staining. The DA neurons in the SNpc were counted using ImageJ and GraphPad software *n* = 3 per group. Unenlarged images scale bars = 200 μm. Image scale bar of the enlarged area of interest = 50 μm. **(C)** Immunofluorescence was used to count CD4^+^ Th17 cells in the SNpc, which were stained for CD4 and IL-17A. The CD4^+^ Th17 cells in the SNpc were counted using ImageJ and GraphPad software *n* = 3 per group. Unenlarged images scale bars = 100 μm. Image scale bar of the enlarged area of interest = 20 μm. Data are presented as the means ± SEMs. ^*^p < 0.05, ^**^p < 0.01, and ^***^p < 0.001.

While peripheral immunity was improved, we observed that the TH content in the SN of the MPTP-injected mice was higher than that in the mice that did not receive PP2 treatment (Figure 6A). The same phenomenon was also observed in the mice treated with MPTP and CCL5. The TH levels in the SN of the mice treated with PP2, MPTP and CCL5 were higher than those in the mice treated with MPTP and CCL5 that did not receive PP2 (Figure 6A). We also observed that pathological α-Syn aggregation in the SNpc was significantly lower in the mice treated with MPTP and CCL5 plus PP2 than in the mice treated with MPTP and CCL5 that did not receive PP2 (Figure 6A). The expression levels of the ICAM-1 protein on the surface of DA neurons, which binds LFA-1 and can induce their death, in the SN of the MPTP-injected mice treated with CCL5 and PP2 were significantly lower than those in the MPTP-injected mice treated with CCL5 that did not receive PP2 treatment (Figure 6A). Furthermore, the number of DA neurons in the SN was higher in the MPTP-injected mice that were treated with PP2 than in the MPTP-injected mice that did not receive PP2 treatment, while the number of DA neurons in the SN was higher in the MPTP-injected mice that were treated with CCL5 and PP2 than in the CCL5-treated MPTP-injected mice that were not treated with PP2 (Figure 6B). In addition, we observed that the number of TH^+^BAX^+^ cells in the SNpc was significantly higher in the MPTP-treated mice than in the MPTP-treated mice treated with PP2 (Supplementary Figure S5A). Similarly, the number of TH^+^BAX^+^ cells in the SNpc was significantly lower in the mice treated with MPTP, CCL5 and PP2 than in the mice treated with CCL5 and MPTP that did not receive PP2 treatment (Supplementary Figures S5A,B). Moreover, in the MPTP-injected mice and MPTP-and CCL5-cotreated mice, the numbers of Th17 cells in the SN were significantly lower with PP2 treatment (Figure 6C).

**Figure 7:**
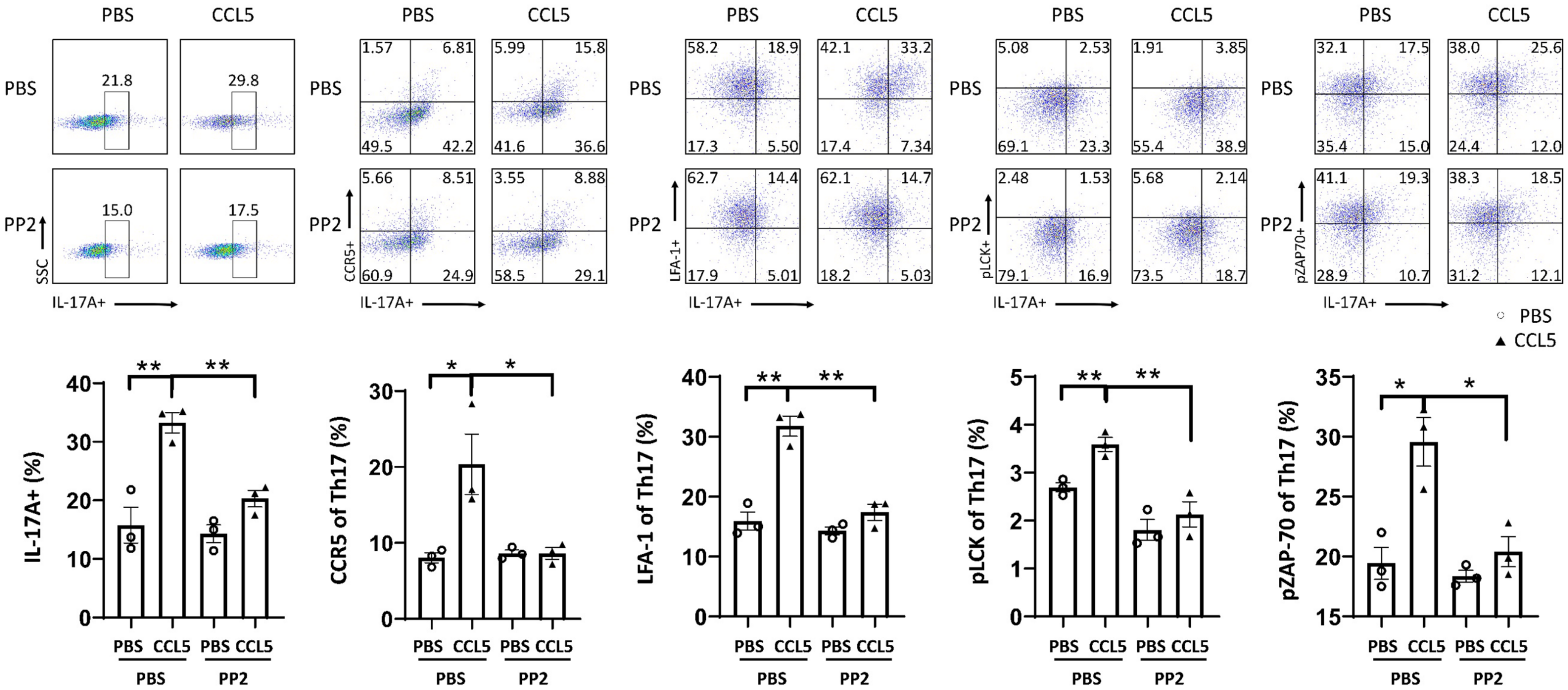
PP2 inhibited Th17 cell differentiation and LFA-1 expression *in vitro*. Naive CD4^+^ T cells from the spleens of C57BL/6J mice were purified by magnetic beads *in vitro* and induced to differentiate into Th17 cells. CCL5 and PP2 were used for treatment. After 3 days, flow cytometry and FlowJo software were used to analyze the cells. The proportions of IL-17A^+^, IL-17A^+^ and CCR5^+^, IL-17A^+^ and LFA-1^+^, IL-17A^+^ and pLCK^+^, and IL-17A^+^ and pZAP70^+^ cells among CD4^+^ cell populations were calculated *n* = 3 per group. Data are presented as the means ± SEMs. ^*^p < 0.05 and ^**^p < 0.01.

We also verified the effect of PP2 on isolated naive CD4^+^ T cells by magnetic bead sorting. *In vitro* culture of Th17 cells differentiated by naive CD4^+^ T cells also demonstrated that PP2 significantly inhibited the phosphorylation of LCK and ZAP70. PP2 critically inhibited CCR5 and LFA-1 expression on Th17 cells and Th17 cell differentiation (Figure 7). These results suggested that the LCK and ZAP70 proteins played a key role in the pathways underlying the effects of CCL5, which involve inducing expression of the LFA-1 protein on Th17 cells and promoting differentiation of naive CD4^+^ T cells into Th17 cells.

Together, these results suggest that CCL5 promotes LFA-1 protein expression in Th17 cells and the differentiation of Th17 cells by activating the LCK and ZAP70 proteins, which can facilitate the migration of Th17 cells from the periphery to the SNpc. This increase in the infiltration of the SNpc by Th17 cells can lead to increased neuroinflammation in DA neurons, increase the loss of DA neurons, and accelerate the progression of PD.

## 4. Discussion

PD is considered an autoimmune disease by an increasing number of researchers due to its pathogenesis and associated pathological changes (Tan et al., 2020; Grotemeyer et al., 2022). Moreover, neuroimmunity is closely related to the pathological progression of PD, and an increase in the number of Th17 cells in the SNpc is an important factor involved in DA neuron injury and loss. In this study, we found that the cytokine CCL5 increased not only the proportion of peripheral Th17 cells in PD mice but also the expression of the LFA-1 protein on the surface of Th17 cells by activating LCK and ZAP70, which facilitated the infiltration of Th17 cells into the SNpc. Moreover, the number of Th17 cells in the SNpc and the number of DA neurons that died were significantly increased in CCL5-treated PD mice. However, when LCK and ZAP70 activation was inhibited, the proportion of peripheral Th17 cells was decreased, and LFA-1 expression on Th17 cells was significantly reduced. Moreover, the number of Th17 cells in the SNpc was significantly reduced, and DA neurons were substantially protected.

Th17 cells are an important component of the pathogenesis and progression of PD (Siffrin et al., 2010; Chen et al., 2015; Pikor et al., 2015; Chen et al., 2017; Liu et al., 2017; Yang et al., 2017; Sommer et al., 2018; Prots and Winner, 2019). The proportion of Th17 cells has been shown to be increased in the peripheral blood of PD patients (Chen et al., 2015, 2017; Yang et al., 2017; Sommer et al., 2018) and in the SN of MPTP-injected mice (Reynolds et al., 2010). These increased proportions of Th17 cells in the peripheral blood and in the SN lead to the destruction of peripheral immune homeostasis and neuroinflammation in the SN, leading to the death of DA neurons in the SN and the aggravation of PD. We found that the proportion of Th17 cells in the spleen of MPTP-injected PD model mice was increased, the number of Th17 cells in the SN was significantly increased, and the loss of DA neurons was also increased. Moreover, as the number of Th17 cells in the SNpc increased, the number of dead DA neurons also increased. Combined with the above discussion, we also hope to determine how MPTP causes an increase in the proportion and number of Th17 cells in the peripheral blood and in the SN of mice. As a common drug used in the modeling of PD in mice, MPTP can be converted into 1-methyl-4-phenylpyridinium (MPP) in mice and can destroy the blood-brain barrier (BBB), damage the mitochondria of DA neurons specifically, and induce the release of reactive oxygen species (ROS), resulting in the death of DA neurons in the SN of mice. It further triggers the body’s subsequent response to the death of DA neurons, including Th17 cells infiltrating into the SN (Javitch et al., 1985; Reynolds et al., 2010). However, the specific mechanism of Th17 infiltration into the SN has not been fully elucidated, and further studies are still needed. These results and articles suggest that the increase in the number of Th17 cells in the SN is essential for the progression of PD. This process may be an important target in the treatment of PD by preventing an increase in the number of peripheral Th17 cells and reducing their infiltration of the SN.

The levels of the chemokine CCL5 were observed to be increased in the blood of PD patients (Rentzos et al., 2007; Tang et al., 2014). Moreover, CCL5 levels were reported to be positively correlated with the severity of PD (Tang et al., 2014). However, CCL5 was shown to have no effect on DA neuronal loss in MPTP-challenged Rag1^−/−^ mice, which do not have T cells (Dutta et al., 2019). Additionally, CCL5 has been proven to specifically induce Th17 cells to migrate and infiltrate the SN, leading to the death of DA neurons and the aggravation of PD (Dutta et al., 2019). Therefore, CCL5 may indirectly affect DA neurons by affecting Th17 cells, leading to the death of DA neurons and the aggravation of PD. Our study also demonstrated that CCL5 not only induced an increase in the number of Th17 cells in the SN but also promoted differentiation and increased the proportion of peripheral Th17 cells. Consequently, these effects of CCL5 on Th17 cells significantly increased the loss of DA neurons in the SN and promoted behavioral deficits in PD mice, resulting in the aggravation of PD symptoms.

Interestingly, we observed that although CCL5 could increase the proportion and number of Th17 cells in the peripheral blood and in the SN of mice when used alone, it did not significantly affect the number or function of DA neurons in the SN of mice. When CCL5 and MPTP are applied together, CCL5 can further promote the death of DA neurons in the SN of MPTP mice. We speculate that the reasons may be as follows. MPTP causes the death of DA neurons in the SN (Javitch et al., 1985), which leads to neuroinflammation in the SN. CCL5, a well-known immunoinflammatory chemokine, can promote the migration of Th17 cells to the SN, and the destruction of the BBB makes this process easier, which further aggravates the inflammatory response of DA neurons in the SN and the death of DA neurons and further promotes the progression of PD pathology. Therefore, we suspect that CCL5 is one of the factors that further promotes the death of DA neurons in mice rather than causing DA neuron death and PD. CCL5 plays a role only after the occurrence of immune inflammation, and the increased death of DA neurons and the exacerbation of immune-inflammation caused by CCL5 further promote this function of the protein. In addition, the death of DA neurons caused by MPTP may promote the production of CCL5 and thus promote the migration of Th17 cells to the SN, which may also be one of the mechanisms of Th17 cell migration to the SN from the peripheral blood of MPTP mice. This remains to be verified by our further experiments.

CCL5 has been proven to promote the migration of T cells outside blood vessels because it promotes the expression of LFA-1 on T cells (Steiner et al., 2010; Evans et al., 2011). Additionally, Th17 cells have been shown to penetrate CNS with the help of the LFA-1 protein in experimental autoimmune encephalomyelitis (EAE) (Rothhammer et al., 2011). Moreover, the LFA-1 protein on the surface of Th17 cells is believed to bind ICAM-1 on the surface of DA neurons to promote the death and loss of DA neurons in PD (Liu et al., 2017). Similarly, the results of our study also indicated that CCL5 significantly increased the expression of the LFA-1 protein on the surface of Th17 cells, and CCL5-treated MPTP-injected mice showed more Th17 cell infiltration and more DA neuronal loss in the SN. However, when LFA-1 expression was inhibited, the number of Th17 cells that had infiltrated the SN was significantly reduced in both CCL5-treated and untreated MPTP-injected PD model mice, and DA neurons were protected. These findings indicated that the migration and infiltration of Th17 cells into the SNpc was completed with the assistance of LFA-1, and the migration ability of Th17 cells was significantly reduced when LFA-1 expression was inhibited.

The roles of Th17 cells in PD neuroimmunity cannot be ignored, and LFA-1 is indispensable for the migration of Th17 cells. We found that CCL5 induced LFA-1 protein expression in Th17 cells, which aggravated PD progression. Therefore, we found that CCL5 promotes neuroimmune progression in PD. However, the specific mechanism underlying CCL5-induced LFA-1 expression in Th17 cells in PD has not been clarified. ZAP70 and LCK are key proteins involved in the expression of the LFA-1 protein in T cells (Dutta et al., 2017; Au-Yeung et al., 2018). We also found increased levels of phosphorylated and activated LCK and ZAP70 in CCL5-stimulated naive CD4^+^ T cells. When the phosphorylation of LCK and ZAP70 was inhibited, the expression of the LFA-1 protein on the surface of Th17 cells was significantly reduced, and CCL5-treated MPTP-injected mice that were treated with PP2 exhibited a reduction in the number of Th17 cells in the SN, a reduction in the number of lost DA neurons, and significant improvements in behavioral deficits, with a significant reduction in PD symptoms. These results suggested that the phosphorylation and activation of LCK and ZAP play a key role in the expression of LFA-1 in Th17 cells.

Furthermore, LCK and ZAP70, which are signaling proteins that function downstream of TCR proteins, also play important roles in the differentiation and activation of T cells (Cridge et al., 2006). Phosphorylated LCK and ZAP70 can activate the expression of *Rorc* genes and promote the secretion of the cytokine IL-17A by activating downstream LAT, PIP2 and other proteins, thus promoting the differentiation of Th17 cells (Cridge et al., 2006). In our study, CCL5 stimulation did not increase the proportion of Th17 cells differentiated from naive CD4^+^ T cells when the phosphorylation and activation of LCK and ZAP70 were inhibited. This suggests that pLCK and pZAP70 activated by CCL5 in naive CD4^+^ T cells can also participate in the TCR pathway, which could promote Th17 cell differentiation.

CCL5 can reduce the efficiency of TCR transport to the T-cell surface (Shaik et al., 2019). However, our experiments demonstrated that CCL5 did not affect the amount of TCRs on the Th17 cell surface but activated the phosphorylation of LCK and ZAP70 to promote the expression of LFA-1. Therefore, based on our experimental results, we speculated on the possible reason for this observation. Studies have shown that the CCL5 receptor CCR5 is coupled with the molecule CD4 (Xiao et al., 1999; Kim et al., 2003), and a zinc clasp structure tethers LCK to the T-cell coreceptor CD4 (Kim et al., 2003). We hypothesize that CCL5 can transmit the signal to CD4 by stimulating CCR5 and then induce LCK activation and phosphorylation. In this way, CCL5 activates the phosphorylation of LCK, which in turn activates the phosphorylation of downstream ZAP70 and LFA-1.

Taken together, the results of this study suggest that CCL5 promotes the differentiation of Th17 cells and the expression of LFA-1 by promoting the phosphorylation of LCK and ZAP70 in PD, which is conducive to the migration and infiltration of Th17 cells into the SN and causes the loss of DA neurons, aggravating the onset and progression of PD.

## 5. Conclusion

**Figure 8:**
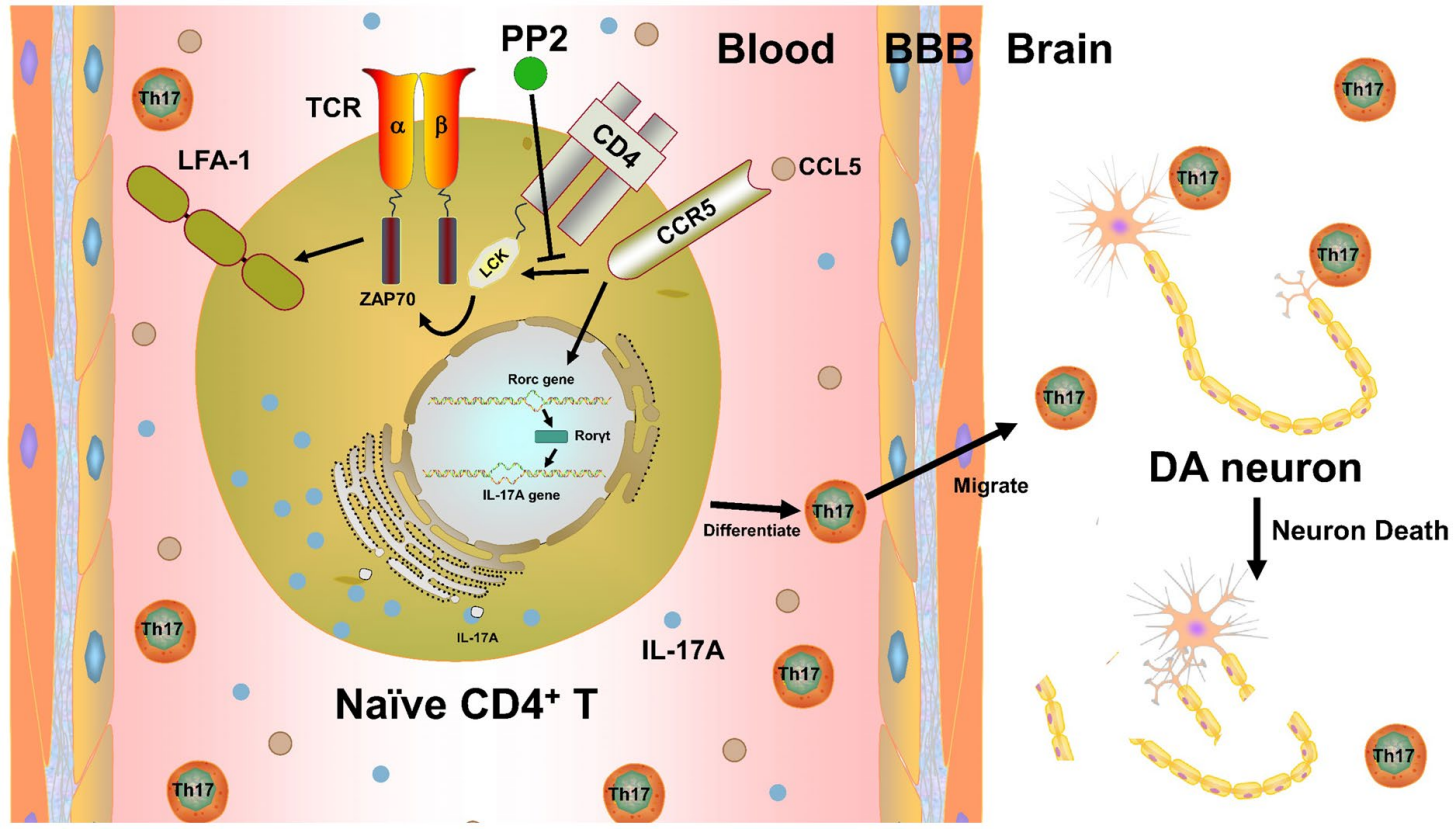
CCL5 affected Th17 cell differentiation and function in PD by activating LCK and ZAP70 in Th17 cells. CCL5 activates LCK and ZAP70 by stimulating the Th17 cell surface receptor CCR5, which in turn promotes the expression of LFA-1 on the Th17 surface and increases Th17 differentiation. The influence of CCL5 on Th17 cells can promote an increase in the Th17 cell proportion and infiltration into the SNpc, induce neuroinflammation and death of DA neurons, and participate in PD-related pathology.

In summary, we propose a novel mechanism by which CCL5 promotes the differentiation of Th17 cells and the expression of LFA-1 protein on the surface of Th17 cells in mice with PD by activating LCK and ZAP70 proteins in Th17 cells, leading to the infiltration of the SNpc by Th17 cells and inducing neuroinflammation in DA neurons and their death (Figure 8). Our study thus demonstrates a critical role for LCK and ZAP70 in the regulation of Th17 differentiation and function stimulated by CCL5 and PD pathologies and may offer novel therapeutic strategies for PD.

## Data availability statement

The original contributions presented in the study are included in the article/Supplementary material, further inquiries can be directed to the corresponding authors.

## Ethics statement

The animal study was approved by Animal Ethics Committee of Tongji Hospital. It is under the Hubei Animal Ethics Committee. The study was conducted in accordance with the local legislation and institutional requirements.

## Author contributions

JZ was responsible for all experiments, data analysis, and writing the manuscript. ZMa and DW provided assistance in the use of relevant flow cytometry software. YQ and JL provided technical assistance in the western blot experiments. KA provided technical support, such as sacrificing the mice and extracting tissue samples. DW, YQ, and JL helped design the experiments. ZX and ZMi contributed to the study design and paper preparation. All authors contributed to the article and approved the submitted version.

## Figures and Tables

**Table 1:** 

Α-Syn	Alpha-Synuclein
AIDS	Acquired Immune Deficiency Syndrome
BBB	Blood-Brain Barrier
CCL5	C-C Chemokine Ligand 5
CNS	Central Nervous System
DA	Dopaminergic
EAE	Experimental Autoimmune Encephalomyelitis
FBS	Fetal Bovine Serum
HIV	Human Immunodeficiency Virus
LCK	Lymphocyte-Specific Protein Tyrosine Kinase
LFA-1	Lymphocyte Function-Associated Antigen-1
MPP	1-Methyl-4-Phenylpyridinium
MPTP	Methyl-4-Phenyl-1,2,3,6-Tetrahydropyridine
PD	Parkinson’s Disease
RIPA	Radioimmunoprecipitation Assay
ROS	Reactive Oxygen Species
SN	Substantia Nigra
SNpc	Substantia Nigra Pars Compacta
TH	Tyrosine Hydroxylase
ZAP70	Tyrosine Kinase Zeta-Chain-Associated Protein Kinase 70
