# Supplementary material for: CCL5 promotes LFA-1 expression in Th17 cells and induces LCK and ZAP70 activation in a mouse model of Parkinson’s disease
Source: Front Aging Neurosci. 2023 Nov 3;15:1250685. doi: 10.3389/fnagi.2023.1250685 (PMC10655117; doi:10.3389/fnagi.2023.1250685)
Supplement: Supplementary file 1 [file Data_Sheet_1.pdf]

# *Supplementary Material*

## 1 Supplementary Figures and Tables

### 1.1 Supplementary Figures

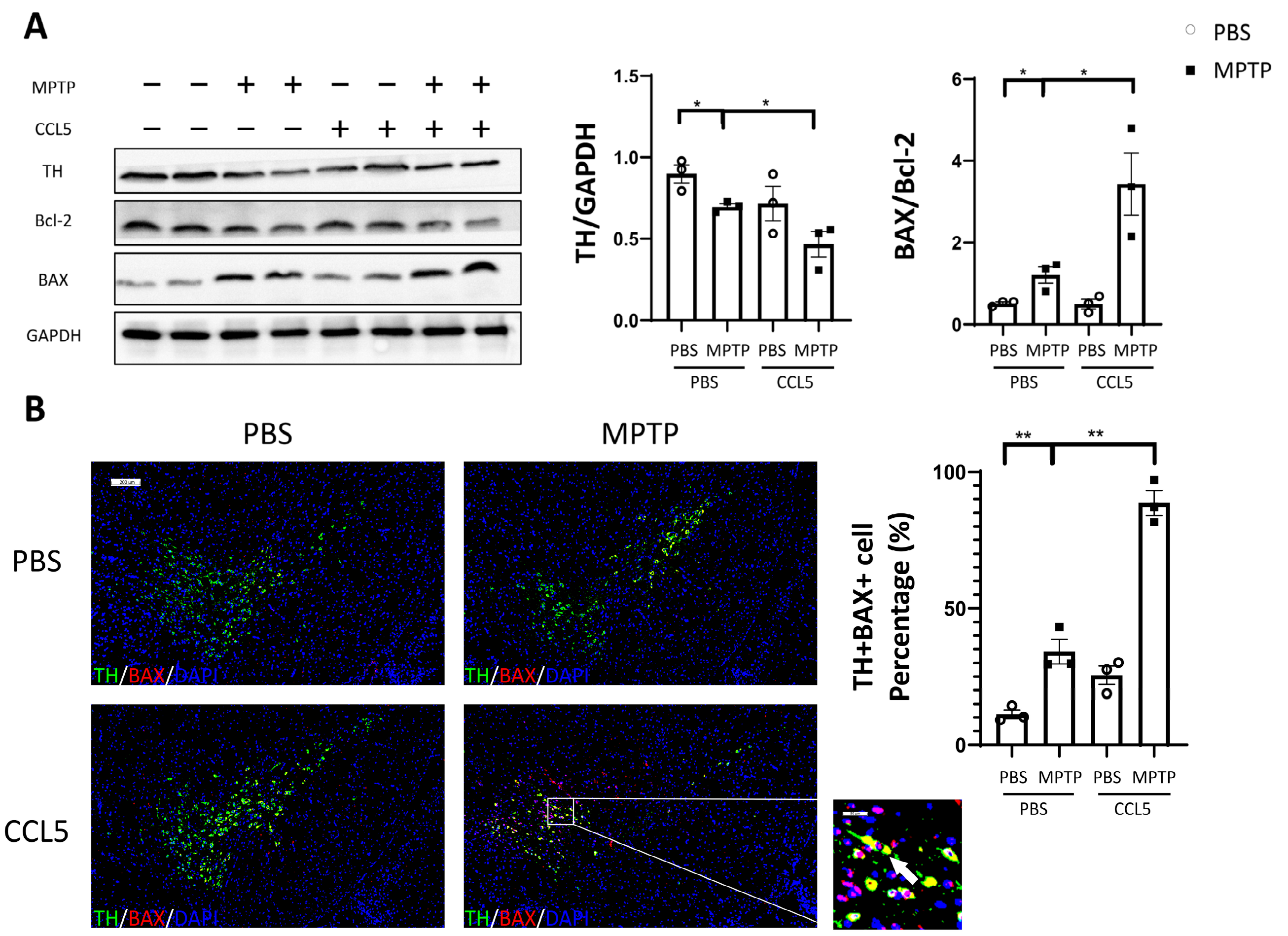


**Fig. S1: CCL5 promoted the expression of apoptosis-related proteins in DA neurons from the mouse SNpc.** (A) Western blot analysis was used to measure TH, BAX and Bcl-2 levels in the brain SNpc of mice, and the data were quantified using ImageJ. n = 3 per group. (B) Immunofluorescence was used to calculate the ratio of $TH^+BAX^+$ cells in the SNpc, which were stained for TH and BAX. A cell was

identified as a $TH^+BAX^+$ cell when both stained TH and BAX were present around the nucleus stained by DAPI and take on a cytoplasmic form that completely or partially envelps the nucleus. While only the stained TH wrapped the nucleus it was identified a $TH^+BAX^-$ cell. The ratio of $TH^+BAX^+$ cells showed the proportion of $TH^+BAX^+$ cells in $TH^+BAX^-$ cells. The arrows within the enlarged area of interest indicate $TH^+BAX^+$ cells in the SNpc, as shown by immunofluorescence staining. The ratio of $TH^+BAX^+$ cells in the SNpc was calculated using ImageJ and GraphPad software. n = 3 per group. Unenlarged images scale bars = 200 μm. Image scale bar of the enlarged area of interest = 50 μm. Data are presented as the means ± SEMs. *$p < 0.05$, **$p < 0.01$.

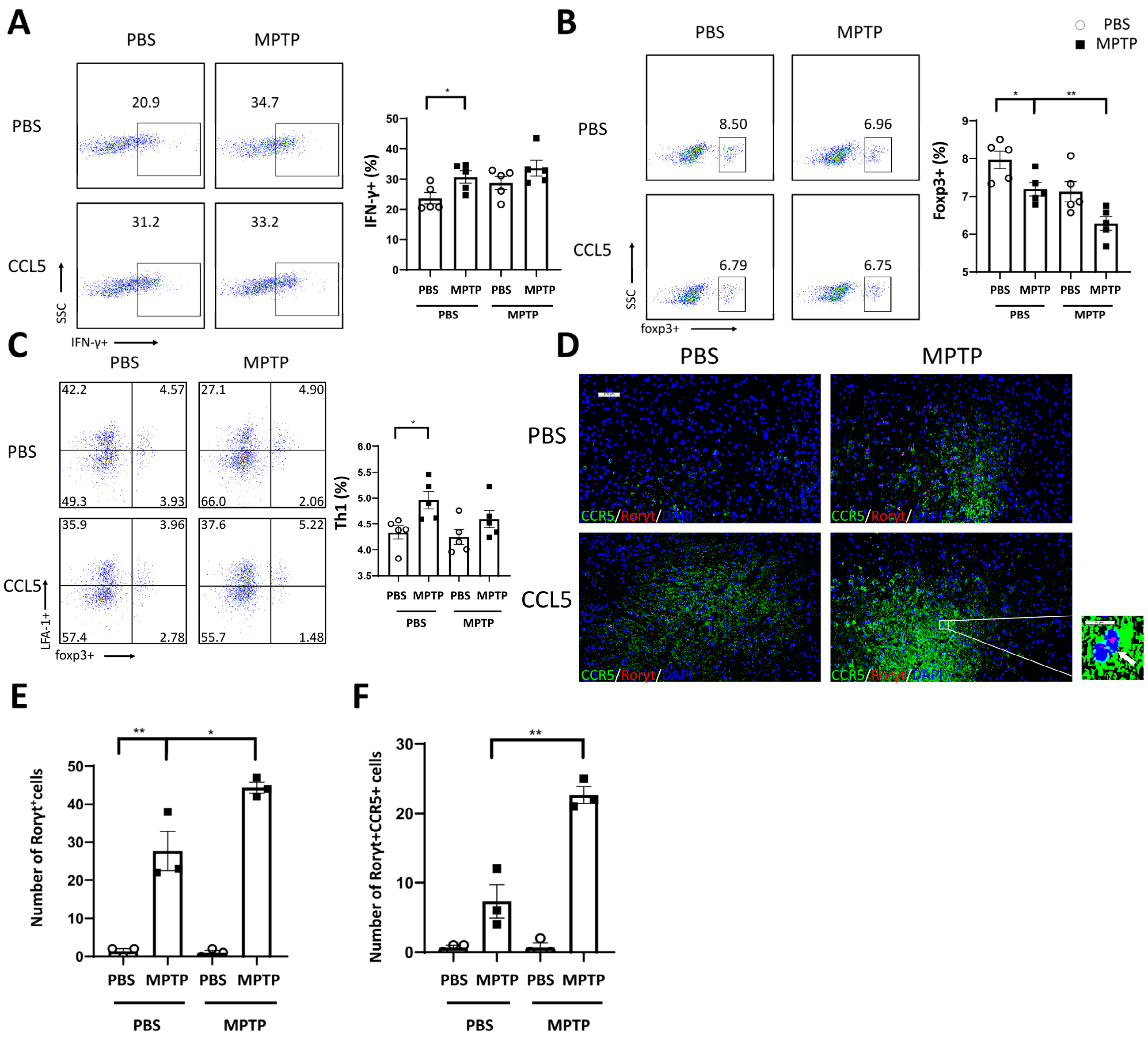
A
PBS
MPTP
PBS
CCL5
20.9
34.7
31.2
33.2
SSC
IFN-γ+
IFN-γ+ (%)
PBS MPTP PBS MPTP
PBS
MPTP
B
○ PBS
■ MPTP
8.50
6.96
6.79
6.75
foxp3+
Foxp3+ (%)
C
42.2
4.57
49.3
3.93
27.1
4.90
66.0
2.06
35.9
3.96
57.4
2.78
37.6
5.22
55.7
1.48
LFA-1+
Th1 (%)
D
CCR5/Rorγt/DAPI
E
Number of Rorγt+cells
F
Number of Rorγt+CCR5+ cells

**Fig. S2: CCL5 could not promote the differentiation of Th1 cells in the mouse spleen or the expression of LFA-1 on Treg cells but could promote the accumulation of CCR5 in Th17 cells in the mouse SNpc at the inflammatory site.** (A, B, C) Flow cytometry and FlowJo software were used to analyze PBMCs from mice. The proportions of IFN-$\gamma^{+}$, Foxp$^{3+,}$ Foxp$^{3+}$ and LFA-1$^{+}$ cells among CD4-positive cell populations were calculated. n=5 per group. (D, E, F) Immunofluorescence was used to count Rorγt$^{+}$CCR5$^{+}$ cells in the SNpc, which were stained for Rorγt and CCR5. A cell was defined when Rorγt is observed in a DAPI-stained nucleus and CCR5 partially or completely wraps the nucleus. The arrows within the enlarged area of interest indicate Rorγt$^{+}$CCR5$^{+}$ cells in the SNpc, as shown by immunofluorescence staining. The Rorγt$^{+}$ cells and Rorγt$^{+}$CCR5$^{+}$ cells in the SNpc were counted using ImageJ and GraphPad software. n = 3 per group. Unenlarged images scale bars = 100 μm. Image scale bar of the enlarged area of interest = 20 μm. Data are presented as the means ± SEMs. *p < 0.05, **p < 0.01.

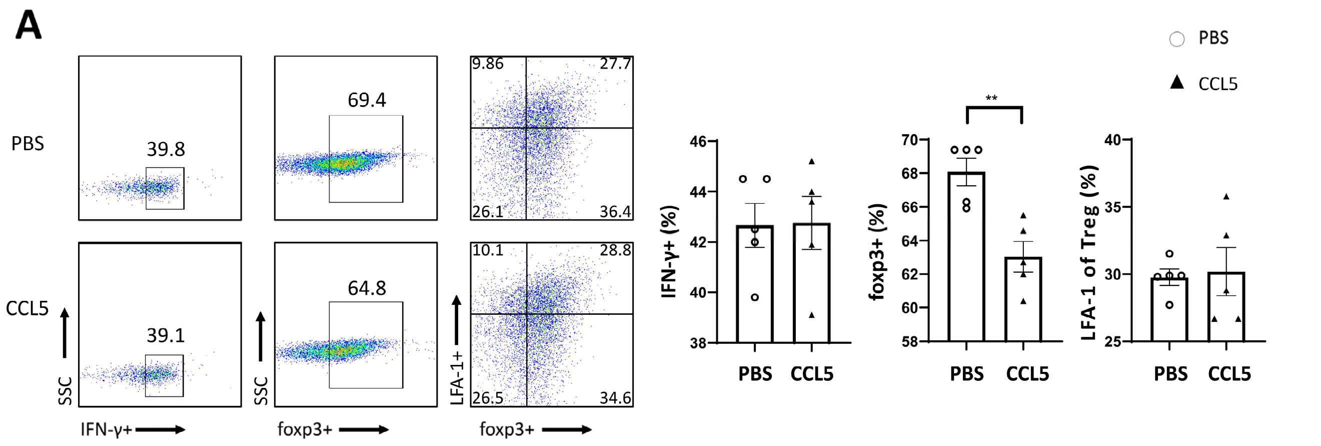


**Fig. S3: Although CCL5 could reduce the ratio of naive $CD4^+$ T cells to Treg cells, it did not affect the ratio of naive $CD4^+$ T cells to Th1 cells or the expression of LFA-1 on the surface of Treg cells.** Naive $CD4^+$ T cells from the spleens of C57BL/6J mice were purified by magnetic beads in vitro and induced to differentiate into Th1 or Treg cells by PBS/CCL5 (1 μg/ml) treatment. After 3 days, the cells were collected. (A) Flow cytometry and FlowJo software were used to analyze the cells. The proportions of IFN-$\gamma^+$, $Foxp3^+$, $Foxp3^+$ and LFA-$1^+$ cells among $CD4^+$ cell populations were calculated. n=5 per group. Data are presented as the means ± SEMs. *$p < 0.05$, **$p < 0.01$.

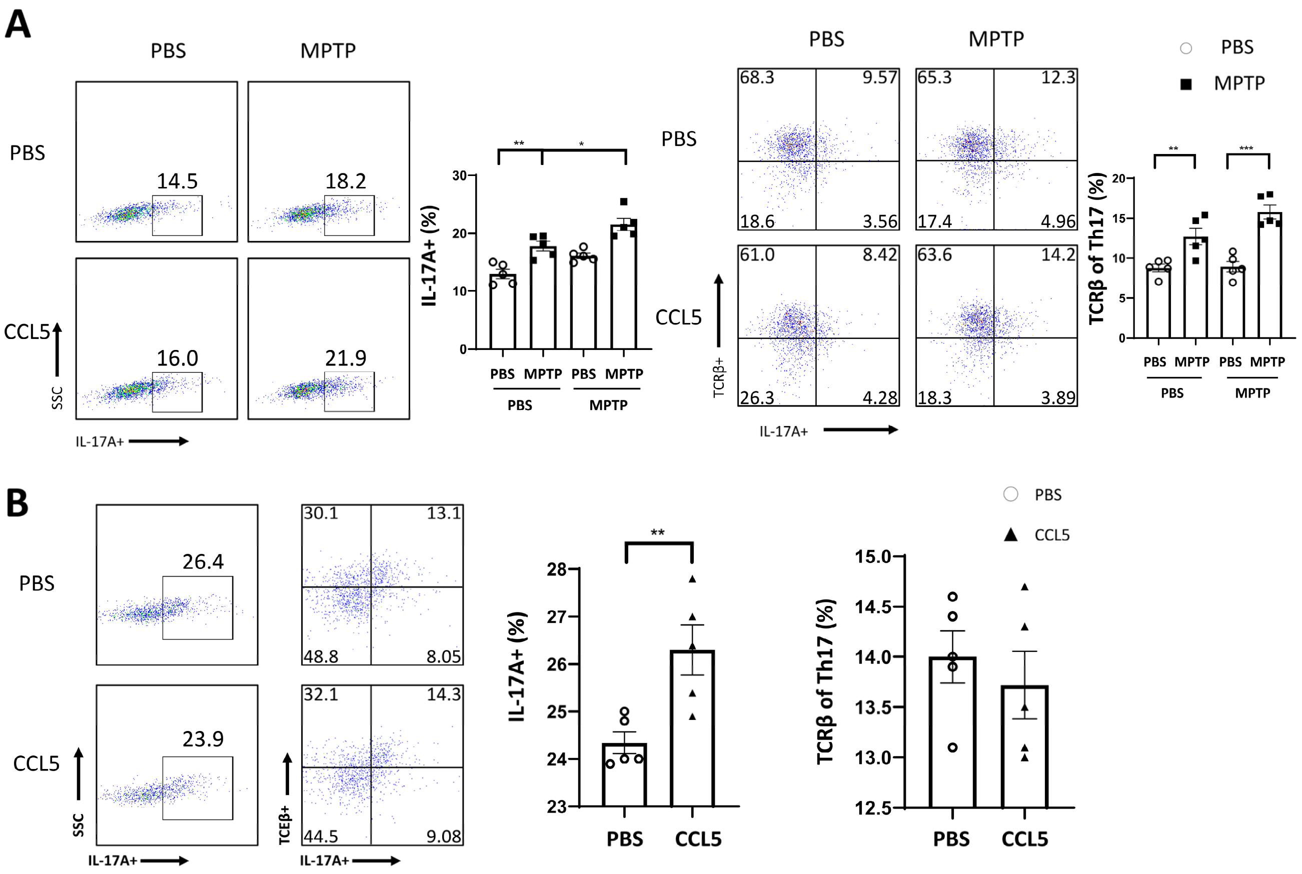


**Fig. S4: The expression ratio of the TCRβ protein on Th17 cells was not affected by CCL5.** (A) Flow cytometry and FlowJo software were used to analyze PBMCs from mice. The proportions of IL-17A$^+$, IL-17A$^+$ and TCRβ$^+$ cells among $^{CD4+}$ cell populations were calculated. n=5 per group. (B) Naive CD4$^+$ T cells from the spleens of C57BL/6J mice were purified by magnetic beads in vitro and induced to differentiate into Th17 cells by PBS/CCL5 (1 μg/ml) treatment. After 3 days, the cells were collected. Flow cytometry and FlowJo software

were used to analyze the cells. The proportions of IL-17A$^+$, IL-17A+ and TCRβ$^+$ cells among CD4$^+$ cell populations were calculated. n=5 per group. Data are presented as the means ± SEMs. *p < 0.05, **p < 0.01, ***p < 0.001.

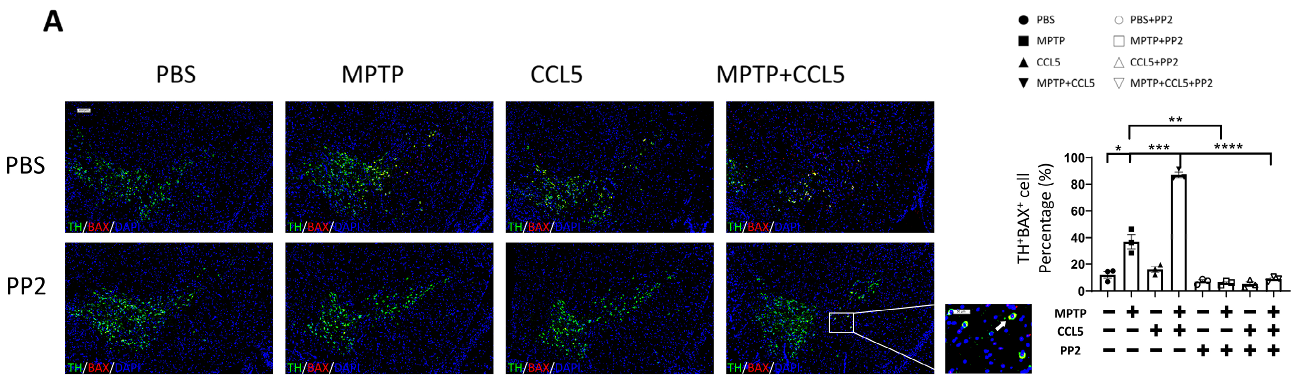


**Fig. S5: PP2 inhibited the expression of apoptosis-related proteins in DA neurons in the SNpc of mice.** (A) Immunofluorescence was used to calculate the ratio of TH$^+$BAX$^+$ cells in the SNpc, which were stained for TH and BAX. The arrows within the enlarged area of interest indicate TH$^+$BAX$^+$ cells in the SNpc, as shown by immunofluorescence staining. The ratio of TH$^+$BAX$^+$ cells in the SNpc was calculated using ImageJ and GraphPad software. n = 3 per group. Unenlarged images scale bars = 200 µm. Image scale bar of the enlarged area of interest = 50 µm.. Data are presented as the means ± SEMs. *p < 0.05, **p < 0.01, ***p < 0.001, ****p < 0.0001.
